# Molecular Dynamics Simulations Guide the Gasification Process of Carbon-Supported Nickel Catalysts in Biomass Supercritical Water

**DOI:** 10.3390/ma17174192

**Published:** 2024-08-24

**Authors:** Yuhui Wu, Liang Wu, Fan Liu, Yue Qiu, Runqiu Dong, Jingwei Chen, Daoxiu Liu, Le Wang, Lei Yi

**Affiliations:** 1 International Institute for Innovation, Jiangxi University of Science and Technology, Ganzhou 341000, China; 6720231596@mail.jxust.edu.cn (Y.W.); 6720221120@mail.jxust.edu.cn (L.W.); 6720220987@mail.jxust.edu.cn (F.L.); 6720220997@mail.jxust.edu.cn (Y.Q.); 6720231599@mail.jxust.edu.cn (R.D.); 9120130127@jxust.edu.cn (D.L.); wang-le@jxust.edu.cn (L.W.); 2 College of Mechanical and Vehicle Engineering, Hunan University, Changsha 410082, China; 3 State Key Laboratory of Multiphase Flow in Power Engineering (SKLMF), Xi’an Jiaotong University, Xi’an 710049, China

**Keywords:** carbon-supported catalyst, molecular dynamics, ReaxFF, biomass pyrolysis

## Abstract

In this study, the Density Functional Theory (DFT) Calculations for Molecules and Clusters—ADF module is employed to model carbon-supported nickel catalysts and lignin monomers, integrating the ReaxFF module to simulate molecular dynamics under supercritical water conditions, with a focus on lignin decomposition reactions. Molecular dynamics models for supercritical water gasification are established under various conditions such as catalyst presence, water molecule quantities, and reaction temperature. By comparing simulation systems under different conditions, the yields of and variations in combustible gases (hydrogen and carbon monoxide) are summarized. Introducing heteroatoms into the lattice of the carbon support can alter the electronic structure within graphene, thereby influencing its electrical and electrochemical properties, increasing the number of active sites, and significantly enhancing electrocatalytic activity. Simulation results indicate that carbon-supported nickel metal catalysts can promote the cleavage of C–C bonds in lignin monomers, thereby increasing the rate of water–gas shift reactions and boosting hydrogen production in the system by 105%. Increasing water molecule quantities favored water–gas shift reactions and hydrogen generation while lowering carbon monoxide formation. Moreover, elevating reaction temperatures led to increased hydrogen and carbon monoxide production rates, which were particularly pronounced between 2500 K and 3500 K. These findings offer crucial theoretical insights for advancing efficient hydrogen production through biomass supercritical water gasification.

## 1. Introduction

In modern industrial production, catalysts play a crucial role. Catalysts significantly accelerate reaction rates by providing alternative reaction pathways that lower the energy barrier and activation energy of chemical reactions [1]. This not only reduces energy consumption and production costs but also enhances reaction efficiency. As we enter the 21st century, catalyst technology has rapidly advanced. Modern catalyst technology encompasses not only traditional metal and metal oxide catalysts but also novel materials such as carbon-supported metal catalysts, molecular sieves [2], and metal–organic frameworks (MOFs). These new catalysts demonstrate significant potential in enhancing reaction efficiency, selectivity, and stability. Furthermore, research on smart catalysts and adaptive control systems is poised to become a future hotspot. Advances in these technologies are expected to enhance the precision and efficiency of chemical reaction control, driving catalyst technology towards greater intelligence and sustainability [3].

However, the development of catalyst technology faces several technical challenges. For instance, the active components of catalysts often include rare or expensive materials, and issues related to the stability of their supply and cost limit the large-scale application and manufacturing of catalysts. In the future, research efforts in catalyst technology will continue to focus on improving reaction efficiency, reducing energy consumption, and minimizing environmental impact. Particularly, developing novel catalysts with high activity, selectivity, low environmental pollution, low cost, and recyclability will be a crucial goal [4].

Currently, mainstream metal catalysts are primarily categorized into two types: precious-metal catalysts and non-precious-metal catalysts. Precious-metal catalysts include platinum (Pt), palladium (Pd), rhodium (Rh), iridium (Ir), and osmium (Os), while non-precious-metal catalysts include iron (Fe), copper (Cu), nickel (Ni), and zinc (Zn), among others [5]. Due to their high cost and limited resource reserves, precious-metal catalysts are constrained in industrial applications [6]. In contrast, carbon-based non-precious-metal catalysts, characterized by their low cost and excellent reaction activity, are poised to serve as excellent alternatives to precious-metal catalysts, demonstrating significant market potential [7].

With increasing emphasis on environmental protection and sustainable development, the innovation direction of catalyst technology has evolved. Green chemistry and atom economy have become crucial considerations in catalyst design [8]. For instance, catalysts that operate efficiently under mild conditions and those capable of effectively converting biomass resources are prominent in this shift [9].

The experimental research of Daqian Wang et al. [10] on biomass steam catalytic gasification for hydrogen production explores the characteristics of biochar, biomass-fast-pyrolysis biochar, and nickel-loaded catalysts. They analyzed the impact of different biochar-loaded nickel catalysts on gasification synthesis gas and found that carbon-supported nickel metal catalysts significantly promote biomass-gasification hydrogen production. Therefore, this study aims to develop a carbon-supported nickel metal catalyst for efficiently converting biomass resources into clean energy hydrogen, reducing catalyst production costs and replacing precious-metal catalysts. The research also analyzes the promotion effect of nickel metal catalysts on hydrogen production in biomass supercritical water gasification simulations, aiming to achieve a higher hydrogen yield and the effective utilization of biomass resources. Lihui Yu et al. [11] investigated the supercritical water gasification of lignin with different molecular sieves (3A, 4A, 5A, 13X, and ZSM-5) catalyzed by Ni. They found that nickel exhibits strong catalytic activity in the supercritical water gasification (SCWG) of biomass. Additionally, the activity and stability of Ni catalysts are influenced by the type and properties of supports. Many supports are used for Ni catalysts in the thermochemical conversion of biomass, including Al_2_O_3_, MgO, zeolites, graphene, and carbon supports. Pooya Azadi et al. [12] studied the catalytic activity and hydrogen selectivity of Ni/α-Al_2_O_3_, Ni/talc, Raney nickel, Ru/C, and Ru/γ-Al_2_O_3_ catalysts in the hydrothermal hydrogen production from lignocellulosic biomass. They found that supported nickel catalysts exhibit high hydrogen selectivity. This simulation analyzes the thermal depolymerization and ring-opening process of lignin from biomass in a supercritical water environment at the molecular level, as well as the influence mechanism of carbon-supported nickel catalysts. It delves into the reaction mechanism and kinetic parameters of supercritical water gasification. The goal is to find more economical and efficient catalyst substitutes, while studying the production pattern of hydrogen and its influencing factors through simulations under different conditions. This will guide the optimization of hydrogen production process parameters, provide technical support for the efficient and clean utilization of biomass, and guide the optimization and improvement of actual experiments and industrial production.

Biomass energy refers to the energy stored in biomass, which is the energy green that plants capture from sunlight and convert into chemical energy through chlorophyll, storing it within their biomass. As a major agricultural nation, China possesses abundant biomass resources. If these resources can be fully utilized and they gradually replace traditional energy sources, it could help alleviate China’s dependence on conventional fossil fuels and improve its energy structure. This measure will contribute to achieving China’s dual carbon goals of peaking carbon emissions by 2030 and achieving carbon neutrality by 2060 [13].

Currently, biomass resources are predominantly processed through incineration, which is generally inefficient and struggles to effectively utilize large quantities of biomass. Moreover, incineration generates harmful gases that severely pollute the environment and degrade the atmosphere. Therefore, there is an urgent need to develop efficient biomass resource processing technologies [4]. The biomass supercritical water gasification hydrogen production technology is considered to have enormous potential for biomass resource utilization due to its clean and efficient hydrogen production capabilities [14].

Alkaline catalysts such as potassium hydroxide and potassium carbonate are commonly used in biomass supercritical water gasification processes. Traditional alkaline metal catalysts effectively promote the water–gas shift reaction but face issues such as difficult recovery, large quantities required, susceptibility to scaling, and blockage [15]. While higher concentrations of alkali additives aid in obtaining purer hydrogen, they also escalate costs. Hence, the development of inexpensive and recyclable novel metal catalysts is paramount to enhancing the efficiency and hydrogen yield of biomass supercritical water gasification, thereby driving efficient utilization of biomass energy [16].

Currently, most researchers are primarily focused on exploring aspects of biomass supercritical water gasification for hydrogen production, such as feedstock selection, various reactor types, and different reaction temperature and pressure conditions [17]. However, there is relatively less attention given to the specific impacts of metal catalysts in supercritical water gasification experiments and their reaction processes and mechanisms at the molecular level.

Due to the stringent experimental conditions, long cycles, and high costs associated with supercritical water gasification, existing experiments mainly analyze product outcomes from a macroscopic perspective, making it challenging to observe and analyze the catalytic mechanisms and specific reaction processes at the molecular level. To address this gap, molecular dynamics simulations using the ReaxFF module in the Amsterdam Modeling Suite 2022 (AMS) simulation software can simulate the catalytic promotion of carbon-supported nickel metal catalysts under complex conditions in biomass supercritical water gasification [18].

This simulation method enables the molecular-level analysis of the pyrolysis and ring-opening processes of lignin in biomass in a supercritical water environment and the catalytic effects of the catalyst. The goal is to identify economically viable and more efficient catalyst alternatives. By simulating different conditions (such as varying water molecule quantities, presence of catalyst, temperature, etc.), the research aims to study hydrogen production patterns and influencing factors, optimizing reaction processes to enhance hydrogen yield and efficiency [19]. These findings are expected to guide improvements and optimizations in practical experiments and industrial production processes.

## 2. The Simulation Method

### 2.1. Simulation Software and Modules

Molecular dynamics (MD) is a computational physics method that has evolved from its initial applications in simple systems to simulating complex systems containing millions or even billions of particles, driven by enhanced computing power and algorithmic optimizations. The applications of molecular dynamics have expanded from physics and chemistry to encompass multiple disciplines such as biology, materials science, and drug design. This method numerically simulates the time evolution of atoms or molecules under a specified potential function to investigate the microstructure and macroscopic properties of materials [20].

In molecular dynamics simulations, the state of the system is described by the positions and velocities of particles. The first step in simulation is to determine the initial configuration and velocities of the system, typically based on experimental data or quantum chemical computation results. Subsequently, the system evolves in time according to the interaction potential function and initial conditions. Interactions between particles are described by the potential energy function, and the choice of potential energy function is crucial for the accuracy of simulation results. The Newtonian equations of motion form the foundation for describing particle movement in molecular dynamics. Due to the typically nonlinear nature of these equations and the involvement of a large number of particles, numerical methods are employed for their solution.

In molecular dynamics simulations, the ADF and ReaxFF modules within the Amsterdam Modeling Suite 2022(AMS) software are utilized to simulate chemical reaction processes. The ReaxFF method, which simulates atoms as the basic unit, describes the formation and breaking of atomic bonds based on bond orders derived empirically from distances between interacting atom pairs. The total system energy is represented as follows:

E_system_ = E_bond_ + E_over_ + E_under_ + E_val_ + E_penl_ + E_tors_ + E_conj_ + E_vdWaals_ + E_Coulomb_

In the equation, E_system_, E_bond_, E_over_, E_under_, E_val_, E_penl_, E_tors_, E_conj_, E_vdWaals_, and E_Coulomb_ represent the total potential energy of the system, bond energy, over-coordinated bond energy, under-coordinated bond energy, bond angle energy, covalent bond correction energy, dihedral angle energy, conjugation energy, Van der Waals interaction energy, and electrostatic interaction energy, respectively [21].

### 2.2. Simulation Methodology

Firstly, catalysts and cellulose molecules are modeled and optimized using the ADF module. The module parameters are set as Table 1: the task is geometry optimization to minimize molecular energy and optimize its structure. The optimization process is highly reliable, with error control at the level of 0.001 Å. The basis set is DZP, and the density functional chosen is the PBE theory method within the generalized gradient approximation (GGA) functional. Geometric Optimization: In the Amsterdam Modeling Suite input, within the Main window, “Task”→“Geometry optimization” were selected to perform geometric optimization, and then “Optimize lattice” in the Details→“Geometry optimization” panel was checked. The optimization method was selected as “auto.”

Next, the models of cellulose, carbon-supported nickel catalyst, and water molecules were randomly mixed and added to the system using the ReaxFF module. Table 2 shows the ReaxFF parameter settings.The task was set as Molecular Dynamics (MD), with periodic boundary conditions defined as Bulk, a time step of 0.25 fs, and a total of 700,000 steps. Temperature parameters were exclusively set in this study, hence the choice of the NVT ensemble. The ensemble is a thermodynamic concept, including NVT, NVE, and NPT. N represents the number of particles, V represents the “box” volume in a ReaxFF simulation, E stands for the total energy of the system, T represents temperature, and P represents pressure. The NVT ensemble requires the number of atoms to remain constant, which is a basic condition in a ReaxFF simulation. Even when particles collide, and although the number of particles changes, the simulation is still based on the setting of a fixed number of atoms. This only means that we are using different systems before and after the collision, but no matter which system is used, the calculation method is based on the NVT ensemble with a fixed number of atoms. Although the NVT ensemble theoretically allows for temperature changes, in actual algorithm processing, each system at a different temperature is treated as an independent instantaneous equilibrium ensemble. The calculation method within these systems still follows the rules of the NVT ensemble, even if the size of the box and the temperature are preset. This simulation is for a system with fixed volume, particle number, and temperature, and it is used to study the system’s free energy, thus choosing the canonical ensemble (NVT ensemble). In the NVT ensemble, only the Thermostat is set, with the Thermostat set to NHC, the Damping constant set to 100 fs, and the NHC chain length set to 10. The force field CHOFeAlNiCuS.ff was selected for constructing the molecular dynamics system [22].

## 3. Model Construction and Optimization

### 3.1. Carbon Support Model and Nickel Metal Cluster Model

**Figure 1:**
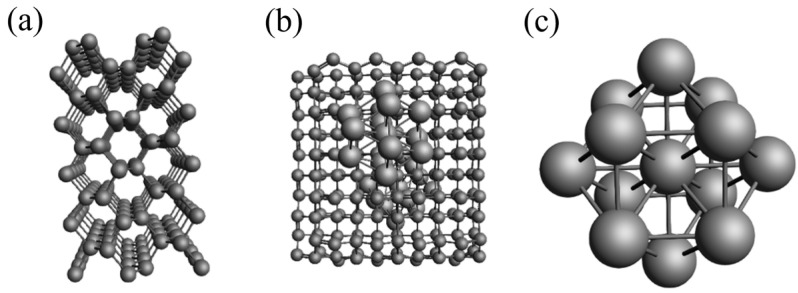
(**a**) Side view of the carbon support model; (**b**) top view of the carbon support model; (**c**) diagram of the Ni cluster model.

**Figure 2:**
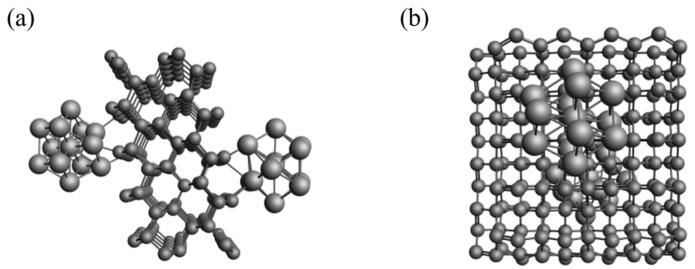
(**a**) Side view of the carbon-supported Ni catalyst model; (**b**) top view of the carbon-supported Ni catalyst model.

In the ADF module, modeling of the carbon support and nickel metal clusters was initiated. Carbon unit cells were imported into the database and transformed into a conventional cell. Using the “Generate slab” option, a supercell of 5 × 5 × 1 was selected with two layers of carbon atoms, forming a periodic model comprising 200 carbon atoms. Subsequently, this model underwent optimization. The C200 model represents the unloaded carbon support, with its specific structure depicted in Figure 1a,b. Figure 1 shows a side view of the carbon support model, while Figure 2 provides a top view. Additionally, a spherical nickel nanocluster model with a radius of 0.3 nm was constructed. The optimized structure, illustrated in Figure 1c, consists of 13 nickel atoms arranged in a cubic octahedral configuration, with Ni–Ni bond lengths ranging from 0.24 to 0.30 nm [23].

### 3.2. Model of Carbon-Supported Nickel Metal Catalyst

The attachment configurations of nickel clusters on carbon support C200 exhibit multiple possibilities. In Configuration 1, four nickel atoms at the base of the cluster interact with four carbon atoms on the support; in Configuration 2, three nickel atoms at the base of the cluster interact with three carbon atoms on the support. This study loaded one Configuration 1 and one Configuration 2 nickel cluster on opposite faces of the carbon support. Subsequently, preliminary optimization was performed using the UFF force field, followed by secondary optimization using Mopic sector. The stabilized configurations after optimization are depicted in Figure 2a,b, with Figure 2a showing a side view of the catalyst model and Figure 2b showing a top view.

### 3.3. Biomass Model

**Figure 3:**
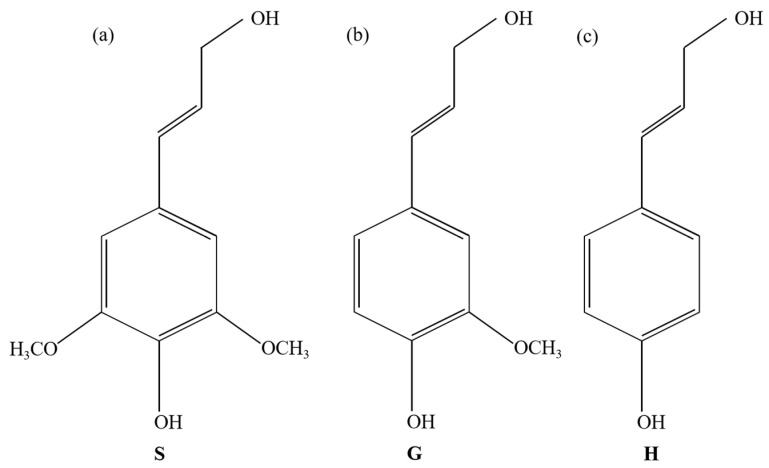
Molecular structures of three types of lignin monomers: (**a**) syringyl lignin; (**b**) guaiacyl lignin; and (**c**) para-hydroxy-phenyl lignin [24].

**Figure 4:**
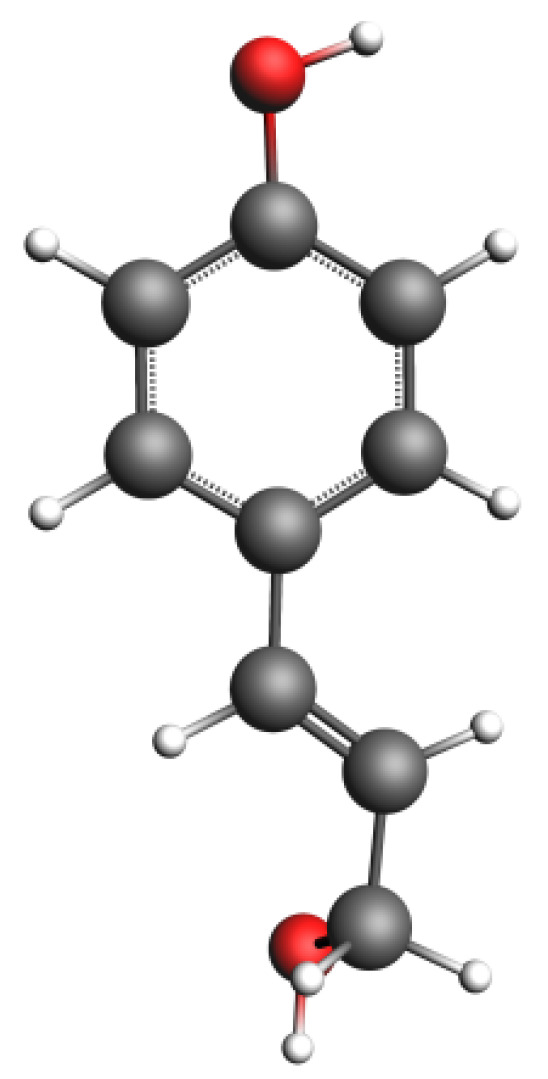
Optimized molecular model of para-hydroxy-phenyl lignin [25].

The focus of this study is on the molecular dynamics simulation of carbon-supported nickel metal catalysts in biomass gasification processes. To achieve this, it is essential to establish a suitable biomass model. Lignin is one of the primary components forming the cell walls of plants, particularly abundant in the hard tissues of wood. Based on differences in monomer structure, lignin can be categorized into three types: syringyl lignin (S-lignin), guaiacyl lignin (G-lignin), and para-hydroxy-phenyl lignin (H-lignin). These three structures are depicted in Figure 3 [23]. For this study, the lignin monomer chosen for the molecular dynamics simulation was para-hydroxy-phenyl propane structure, with its optimized structure shown in Figure 4 [24].

### 3.4. Molecular Dynamics Simulation System

**Figure 5:**
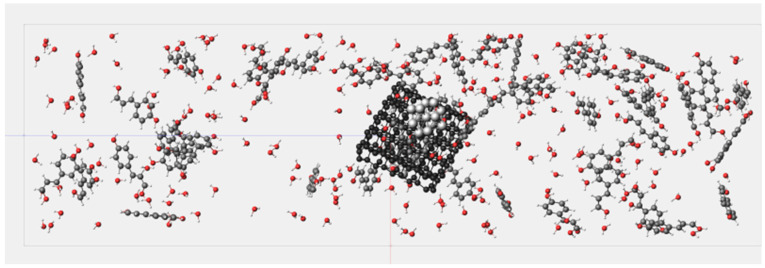
Schematic of supercritical water gasification molecular dynamics simulation System 1.

**Figure 6:**
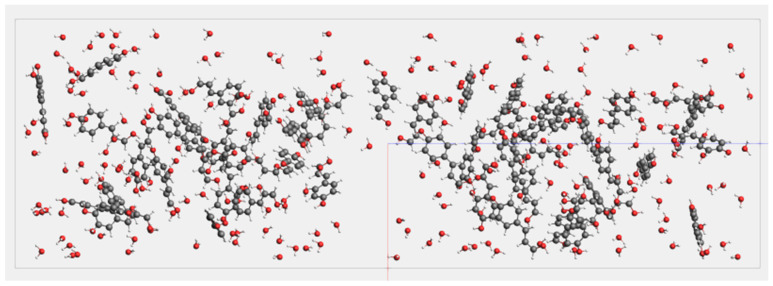
Schematic of supercritical water gasification molecular dynamics simulation System 2.

Based on the foundation laid out earlier, we established the molecular models for the entire molecular dynamics simulation. All models were optimized using the ADF module to ensure optimal structure, bond lengths, and bond angles, thereby avoiding unreasonable structures and minimizing molecular structural energies. Within the ReaxFF module, a cubic structure was constructed with periodic boundary conditions set to Bulk. The lattice vectors parameters of the simulation system were set as follows: a→30 Å, b→30 Å, c→100 Å, resulting in a volume of 90,000.0 Å^3^. The system included 200 water molecule models and 50 lignin molecule models, achieving a system density of 0.2738 g/mL. At this system density, it can be ensured that the system reaches a supercritical state at a simulated temperature. With the overall density known, the concentration of the material is determined, and the density of water can be calculated based on the concentration of the material. Figure 5 and Figure 6 depict two different reaction conditions established by varying the presence of catalyst in the molecular dynamics simulation system. 

## 4. Simulation Results and Discussion

### 4.1. Results of Lignin Gasification

**Figure 7:**
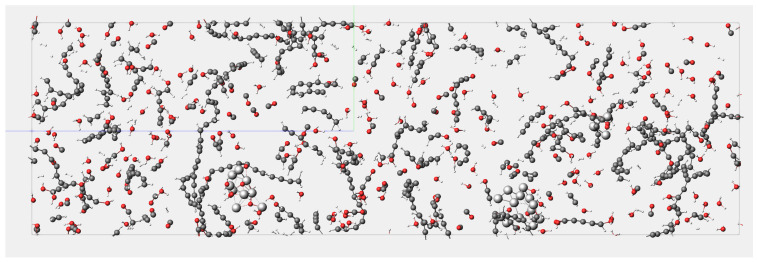
Distribution of reaction products in supercritical water gasification.

**Figure 8:**
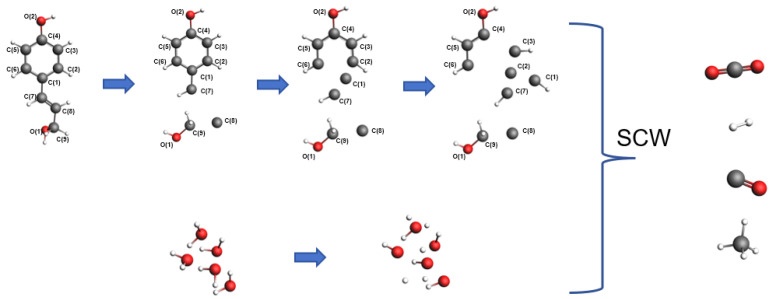
Reaction pathways of lignin in supercritical water gasification.

**Figure 9:**
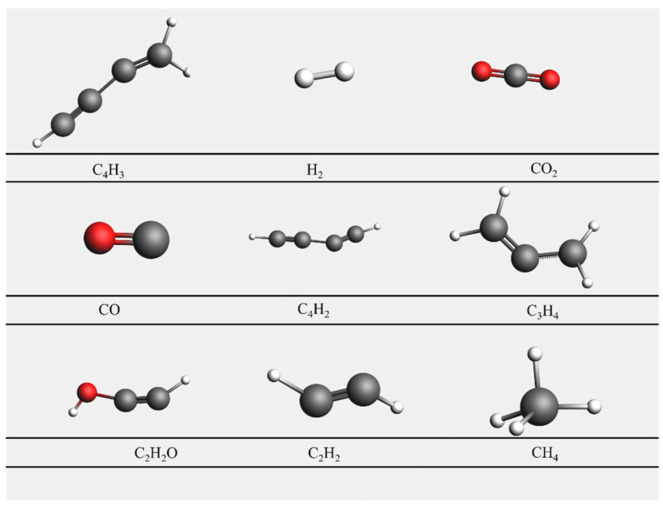
Gasification product diagram.

Figure 7 illustrates the results of the molecular dynamics simulation for System 1 at 3000 K after 7000 frames. It can be observed from the figure that the nickel cluster undergoes fragmentation during the reaction process, dispersing nickel atoms throughout the entire system. Figure 8 depicts the reaction pathways of lignin under supercritical water conditions, including carbon ring-opening, bond cleavage, and transformation into smaller molecular species. Specifically, the C (8) bond in the lignin monomer first breaks, followed by single bond cleavage between C (7) and C (1), leading to the fragmentation of the benzene ring from C (1). Simultaneously, water molecules break apart, generating H and OH radicals that interact with lignin to produce hydrogen, carbon monoxide, carbon dioxide, and methane. Lignin undergoes gasification and pyrolysis in the high-temperature, high-pressure supercritical water environment, resulting in gasification products, as depicted in Figure 9.

**Figure 10:**
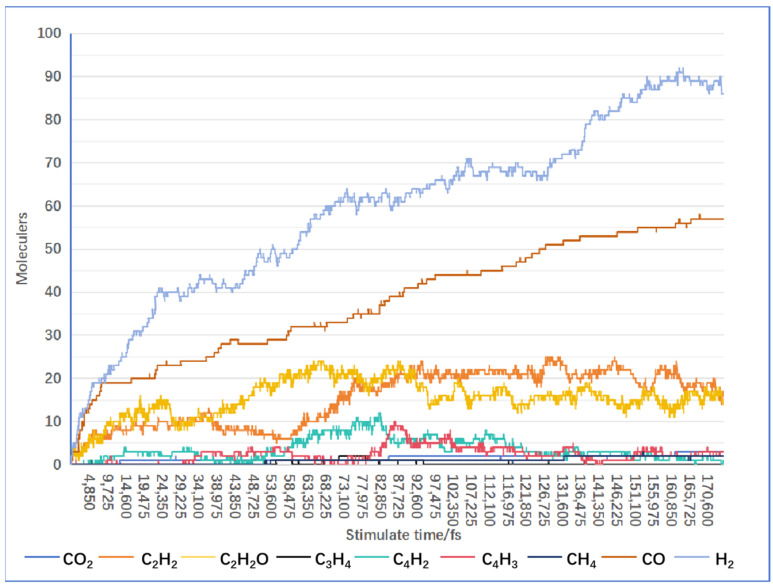
Production rates of major gas products.

By analyzing the composition and generation patterns of products, this study contributes to a deeper understanding of the fundamental principles of the reaction. Specifically, this study focuses on analyzing the gas products of biomass supercritical water gasification. Apart from water molecules inherent to the system, the predominant gas products in the simulation process are H_2_ and CO. Figure 10 illustrates the rate of generation of major gas products from the biomass model lignin during supercritical water gasification. As is shown in Figure 10, the rate of H_2_ generation gradually increases with frame number, with a rapid increase observed in the range of 0 to 4000 frames, stabilizing between 4000 and 7000 frames at approximately 77 to 80 molecules of H_2_. CO exhibits a higher generation rate from 0 to 700 frames, followed by a gradual decrease, reaching a maximum of 72 molecules of CO by 7000 frames.

### 4.2. Effect of Supercritical Water on Gas Product Formation

**Figure 11:**
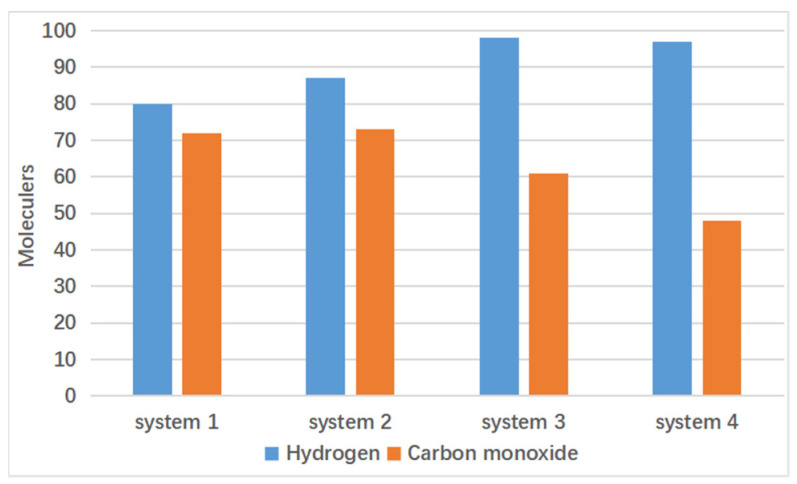
Final combustible gas yields from the four systems.

Four molecular dynamics simulation systems were established: System 1 with 200 water molecules, System 2 with 300 water molecules, System 3 with 400 water molecules, and System 4 with 500 water molecules. The simulation temperature was set at 3000 K, with other conditions kept constant. These systems were used as controls to assess the specific impact of supercritical water on gas products, particularly hydrogen. Figure 11 shows the final combustible gas yields from the four systems.

**Figure 12:**
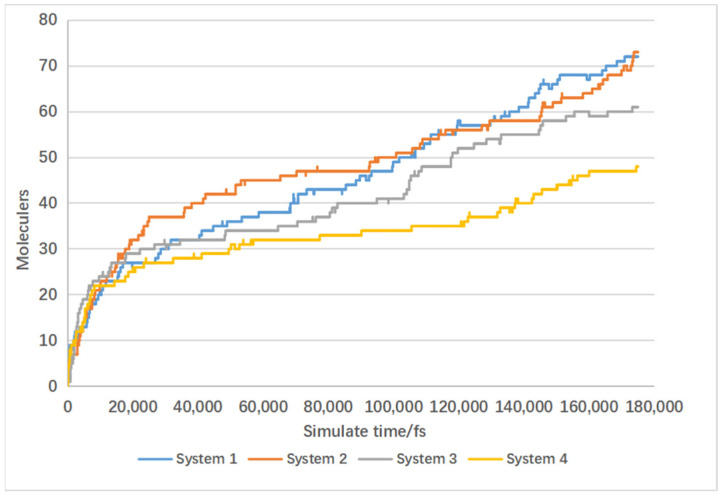
Influence of water molecule quantity on CO production under supercritical conditions.

Figure 12 shows the trends in carbon monoxide production in molecular dynamics simulation Systems 1, 2, 3, and 4. All four systems exhibit a similar pattern: The rate of carbon monoxide production peaks in the first 1000 frames, followed by a gradual slowdown from 1000 to 4000 frames where the production rate stabilizes. From 4000 to 7000 frames, there is a gradual increase again in the rate of carbon monoxide production.

**Figure 13:**
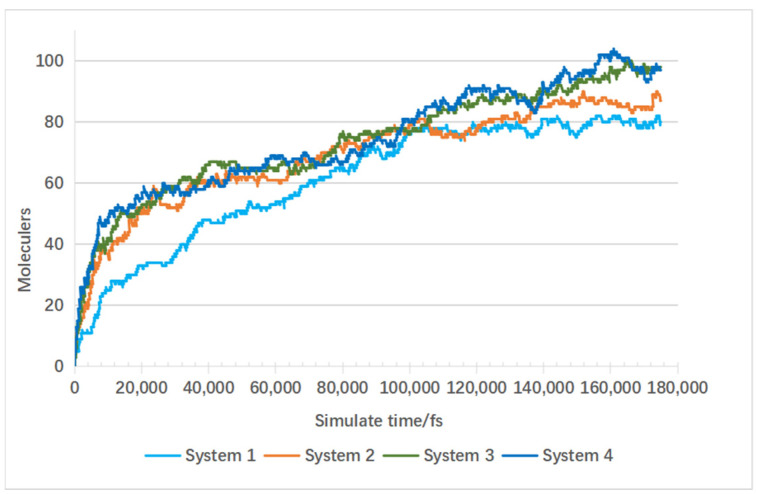
Influence of water molecule quantity on hydrogen production under supercritical conditions.

Among these systems, as the number of water molecules increases, Systems 1 and 2 show similar final counts of carbon monoxide molecules towards the end of the reaction. However, Systems 3 and 4 show a decrease in the final count of carbon monoxide molecules with the increase in water molecules. This indicates that an increase in the number of water molecules leads to a reduction in the final yield of carbon monoxide. This is because water molecules undergo water–gas shift reactions with carbon monoxide, converting it to hydrogen gas, thereby increasing the yield of hydrogen [26]. This conclusion is further supported by Figure 13.

In Figure 13, Systems 2, 3, and 4 exhibit similar overall trends in hydrogen gas growth, while System 1 shows consistently lower overall growth rates compared to the other three systems. The final counts of hydrogen molecules are 98 for System 3, 97 for System 4, 80 for System 1, and 87 for System 2. System 4 shows the highest final hydrogen gas yield, followed by Systems 3 and 2, with System 1 having the lowest hydrogen gas yield.

The results in Figure 12 show that System 1 has the highest carbon monoxide production, followed by Systems 2, 3, and 4 with progressively lower carbon monoxide yields. Thus, the production trends in these two gases are inversely correlated.

From the above results, it can be observed that supercritical water promotes lignin gasification. An increase in the number of water molecules facilitates the water–gas shift reaction, reducing carbon monoxide production and increasing hydrogen gas production.

### 4.3. Effect of Catalyst on Gas Product Formation

Carbon supports act as large molecular ligands in metal catalysts, playing a crucial role in the performance of active sites, thus finding widespread applications in catalyst support fields. Major types of carbon supports include graphene structures, carbon nanotube structures, carbon sphere structures, and multilayer graphene stack structures [27]. In catalyst or support applications, complete multilayer graphene stack structures typically exhibit weaker interactions with active components or additives. Constructing catalyst systems by loading nickel metal atoms onto carbon–carbon-bond supports typically involves fundamental steps such as oxidation addition, transmetallation, and reduction elimination. Nickel involvement in carbon–carbon coupling can sometimes demonstrate unique advantages. Introducing heteroatoms into the carbon support lattice can alter the internal electronic structure of graphene, thereby influencing its electrical and electrochemical properties, increasing the number of active sites, and significantly enhancing electrocatalytic activity [28].

In practical applications, graphene often needs to be modified or doped to enhance its bonding properties with other metal materials. Major modification methods include directly substituting carbon atoms on the single-layer graphene framework, where chemical bonding connects heteroatoms with carbon atoms, offering good stability but potentially introducing defects into the graphene structure, thereby reducing charge carrier mobility [29]. Another approach involves adsorption or doping of heteroatoms or metal clusters onto the surface of carbon supports. While this doping method preserves the integrity of the graphene structure, it may compromise some stability [30].These doping strategies provide important pathways and insights for optimizing the catalytic performance of stacked graphene.

**Figure 14:**
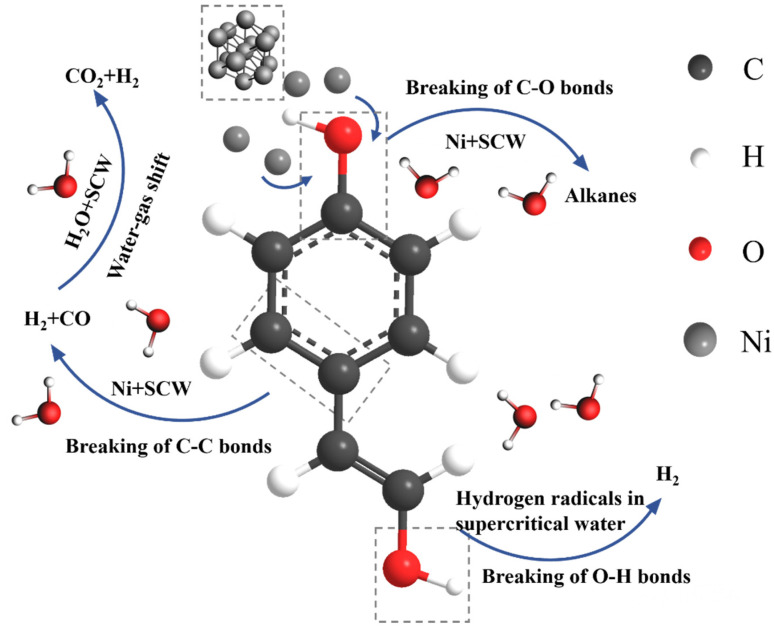
The catalytic mechanism of lignin hydrogenation under nickel catalysts.

**Figure 15:**
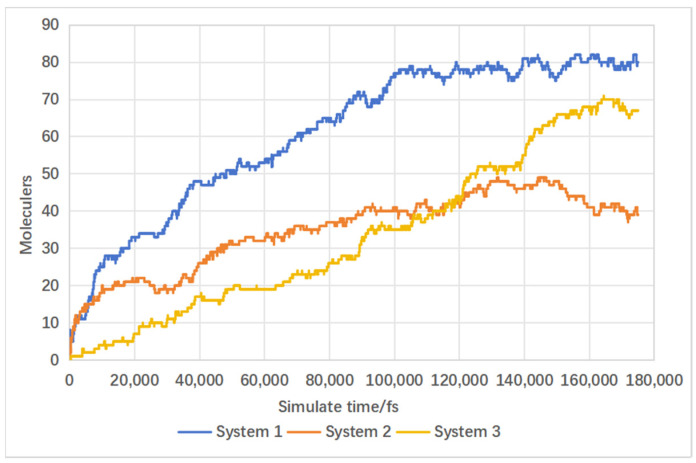
Influence of catalyst on hydrogen production.

**Figure 16:**
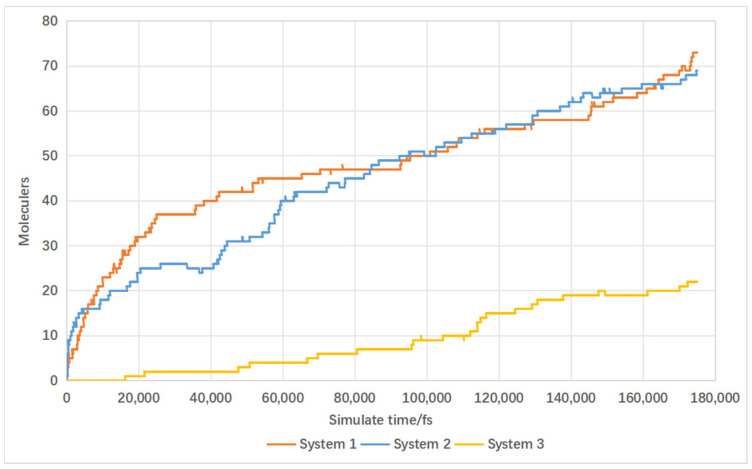
Influence of catalyst on carbon monoxide production.

In this section, three systems were established: System 1 included carbon-supported nickel catalysts, while System 2 incorporated carbon support structures without nickel metal clusters, and System 3 included only the nickel cluster catalyst. serving as a control to verify the catalytic role of nickel metal and the promotional effect of carbon-supported nickel catalysts on supercritical water gasification reactions. Due to the low methane production in the target gas products and the inability of carbon dioxide to burn, this discussion focuses solely on the variations in the production of combustible gases hydrogen and carbon monoxide. Figure 14 depicts the catalytic mechanism of lignin hydrogenation under nickel metal catalysts. Nickel acts on the C–O bonds of lignin monomers, causing their cleavage and the formation of aliphatic compounds under supercritical water conditions, thereby increasing carbon gasification efficiency. Nickel also acts on the C–C bonds of lignin monomer benzene rings, causing their cleavage and the generation of hydrogen and carbon monoxide under supercritical water conditions, thereby enhancing hydrogen production through promoting water–gas shift reactions. As is evident from the hydrogen production curves in Figure 15, the hydrogen generation rate in System 1 with carbon-supported nickel catalysts remained significantly higher throughout, compared to System 2 without nickel metal clusters. The final simulation results showed hydrogen molecule counts of 80 for System 1 and 39 for System 2, indicating a 105% increase in hydrogen yield in System 1. System 1 and System 3 have hydrogen yields much higher than System 2, leading to the conclusion that nickel metal significantly promotes hydrogen production. By observing in Figure 14 that the hydrogen yield of System 1 is higher than that of System 3, it can be inferred that the carbon-supported nickel metal catalyst has a promoting effect on hydrogen production from biomass in supercritical water. It can be concluded that carbon-supported nickel catalysts significantly enhance biomass supercritical water hydrogenation. Figure 16 illustrates the carbon monoxide production rates in both systems, showing similar rates between System 1 and System 2, with little difference in final carbon monoxide molecule counts. Therefore, it can be concluded that the addition or absence of catalysts has no significant effect on carbon monoxide yield.

### 4.4. Effect of Temperature on Gas Production

**Figure 17:**
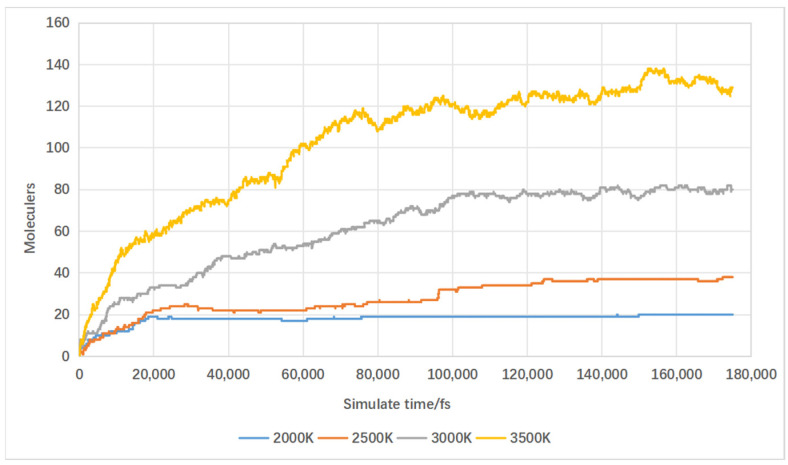
Influence of temperature on hydrogen production.

Temperature is a key factor influencing reaction rates, which is particularly crucial in biomass supercritical water gasification [31]. Low temperatures hinder complete lignin decomposition, while excessively high temperatures lead to the formation of complex compounds containing aromatic hydrocarbons and their derivatives, thereby reducing hydrogen yield [32]. Within the temperature range of this simulation study, increasing the temperature leads to an increase in hydrogen production. The main purpose of this study is to explore the impact of catalysts on the supercritical water gasification process of biomass, with temperature being just one of the variables. Therefore, this paper focuses only on the rate of change in hydrogen within the simulation temperature range. A temperature gradient was set with molecular dynamics simulations conducted under four different conditions: 2000 K, 2500 K, 3000 K, and 3500 K. The simulated results of hydrogen production are shown in Figure 17. Increasing the temperature from 2000 K to 2500 K resulted in a modest increase of 18 hydrogen molecules. Further increasing the temperature from 2500 K to 3000 K boosted hydrogen production by 42 molecules, and from 3000 K to 3500 K, it increased by an additional 59 molecules. This illustrates that higher reaction temperatures enhance hydrogen production, with greater increases observed between 2500 K and 3500 K.

**Figure 18:**
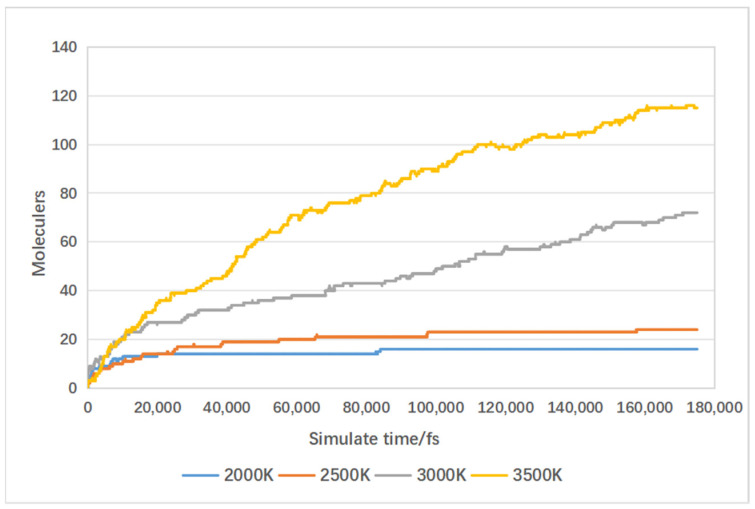
Influence of temperature on carbon monoxide production.

The simulated results of carbon monoxide production are depicted in Figure 18. Increasing the temperature from 2000 K to 2500 K resulted in a slight increase of eight hydrogen molecules. Subsequently, increasing the temperature from 2500 K to 3000 K led to a larger increase of 48 molecules, and from 3000 K to 3500 K, it increased by 43 molecules. This indicates that higher reaction temperatures also enhance carbon monoxide production, with greater increases observed between 2500 K and 3500 K.

## 5. Conclusions

Carbon-supported nickel catalysts significantly enhance hydrogen production in biomass supercritical water gasification, increasing hydrogen yield by 105%, with no significant impact on carbon monoxide production rates. Carbon-supported nickel metal catalysts promote the cleavage of C–C bonds in lignin monomers, thereby increasing the rate of water–gas shift reactions and boosting hydrogen production in the system by 105%.

Temperature plays a promoting role in the supercritical water gasification reaction of biomass. An increase in temperature will accelerate the reaction rate between molecules. In molecular dynamics simulations, as the temperature rises, the production of gaseous products such as hydrogen and carbon monoxide increases. Within the temperature range of 2500 K to 3500 K, the higher the temperature, the higher the yield of hydrogen and carbon monoxide will be. The increase in water molecules provides a broader source of hydrogen radicals, and the breaking of more hydrogen bonds will promote the production of hydrogen. An increase in the number of water molecules in the molecular dynamics simulation system will lead to a decrease in the final yield of carbon monoxide and an increase in the production of hydrogen. This is because water molecules will undergo the water–gas shift reaction with carbon monoxide, converting carbon monoxide into hydrogen, thereby increasing the yield of hydrogen.

## Figures and Tables

**Table 1:** ADF Parameter Settings.

Task	xc Function	Relativity	Basis Set	Frozen Core	Numerical Quality
Geometry Optimization	GGA: PBE	Scalar	DZP	Large	Normal

**Table 2:** ReaxFF Parameter Settings.

Task	Time Step (fs)	Torsions	Periodicity	Number of Steps	Thermostat
Molecular Dynamics	0.25	Original	Bulk	700,000	NHC

## Data Availability

The original contributions presented in the study are included in the article, further inquiries can be directed to the corresponding authors.
